# Coincident frequencies and relative phases among brain activity and hormonal signals

**DOI:** 10.1186/1744-9081-5-18

**Published:** 2009-03-14

**Authors:** Silvia Solís-Ortíz, Rafael G Campos, Julián Félix, Octavio Obregón

**Affiliations:** 1 Departamento de Ciencias Médicas, División de Ciencias de la Salud, Campus León, Universidad de Guanajuato, León 37320, Guanajuato, México; 2 Facultad de Ciencias Físico-Matemáticas, Universidad Michoacana, Morelia, 58060, Michoacán, México; 3 Departamento de Física, División de Ciencias e Ingenierías, Campus León, Universidad de Guanajuato, León 37150, Guanajuato, México

## Abstract

**Background:**

Fourier transform is a basic tool for analyzing biological signals and is computed for a finite sequence of data sample. The electroencephalographic (EEG) signals analyzed with this method provide only information based on the frequency range, for short periods. In some cases, for long periods it can be useful to know whether EEG signals coincide or have a relative phase between them or with other biological signals. Some studies have evidenced that sex hormones and EEG signals show oscillations in their frequencies across a period of 28 days; so it seems of relevance to seek after possible patterns relating EEG signals and endogenous sex hormones, assumed as long time-periodic functions to determine their typical periods, frequencies and relative phases.

**Methods:**

In this work we propose a method that can be used to analyze brain signals and hormonal levels and obtain frequencies and relative phases among them. This method involves the application of a discrete Fourier Transform on previously reported datasets of absolute power of brain signals delta, theta, alpha1, alpha2, beta1 and beta2 and the endogenous estrogen and progesterone levels along 28 days.

**Results:**

Applying the proposed method to exemplary datasets and comparing each brain signal with both sex hormones signals, we found a characteristic profile of coincident periods and typical relative phases. For the corresponding coincident periods the progesterone seems to be essentially in phase with theta, alpha1, alpha2 and beta1, while delta and beta2 go oppositely. For the relevant coincident periods, the estrogen goes in phase with delta and theta and goes oppositely with alpha2.

**Conclusion:**

Findings suggest that the procedure applied here provides a method to analyze typical frequencies, or periods and phases between signals with the same period. It generates specific patterns for brain signals and hormones and relations among them.

## Background

Fourier transform is a basic tool for analyzing biological signals [1-3]. Mostly a fast Fourier transform is computed for finite sequence of data sample [4]. In the case of brain signals obtained from electroencephalographic (EEG) recording, the information can be extracted by spectral analysis, whereby amplitude characteristics of the frequency domain of the EEG signal can be assessed using a technique known as fast Fourier transform, specific frequency bands are identified and the signal amplitude within each of these wavebands calculated for short periods [5,6]. Generally this analysis provides information, according with the frequency range of the well known brain signals delta (1.5 – 3.5 Hz), theta (3.3 – 7.5 Hz), alpha1 (7.5 – 9.5 Hz), alpha2 (9.5 – 12.5 Hz), beta1 (12.5 – 17.5 Hz), beta2 (17.5 – 30.0 Hz) and gamma waves (30–50 Hz), which have been widely used both in clinical and experimental designs [7-11]. However, the EEG signals analyzed with this method provide only information based in frequency range, for short periods. In some cases, for long periods it can be useful to know whether EEG signals coincide or have a relative phase between them or with other biological signals of scientific interest. The endogenous sex hormones are biological signals of interest in many aspects [12], and some studies have evidenced that these signals and EEG signals show oscillations in their frequencies across a period of 28 days [13-19], so it seems of relevance to seek after possible patterns relating with EEG signals and endogenous sex hormones data. In the present paper, we propose one method that can be used to obtain coincidence and relative phases among EEG signals and hormonal levels for long periods. The procedure is established as follows: Assigning certain numerical value, i.e., the absolute power, to each EEG signal at certain sampling times, generates data that can be interpolated through a long period, yielding an absolute power function of time for each signal. A similar procedure is followed in case of hormonal values. A discrete Fourier transform is then performed to analyze these new functions, finding typical frequencies and their corresponding periods for each one of these signals and, also, relative phases for coincident periods between two or more signals. The technique is based on a discrete Fourier transform which gives accurate results even in the case of few point samples [20,21]. We used exemplary dataset from real frequencies EEG recording and values of hormonal levels obtained along of 28 days to illustrate this method. In this way, we found typical frequencies and characteristics phases of the brain signals and of the progesterone and estrogen levels.

## Methods

### Brain signals and hormonal levels

We used a dataset of EEG recording of women and the variations of their estrogen and progesterone levels previously reported [17,22,23]. Briefly, 9 female volunteers aged between 20 and 34 (mean = 27.11; SD = 4.45) participated in the study. They were university students and they were selected on account of their regular cycles (28 days ± 2). None of them were under any type of medication nor were taking or have taken oral contraceptives. A total of 10 sessions of the EEG activity were recorded, 3 times a week every second day, except Sundays, at the same time of the day (between 16 and 19 hrs), at rest with eyes open. EEG recordings started at the subject own convenience, thus the day of menstruation of different subjects were randomly distributed over the sessions. Electrodes were placed according to the 10–20 International System al F3, F4, C3, C4, P3, P4, O1 and O2, referred to ipsilateral earlobes. EEG was recorded on a Grass model 16E polygraph set to pass frequencies between 1 and 35 Hz. Impedance was kept below 10 k 0 hms. EEG was captured in a PC computer using the program CAPTUSEN [24], by a 12-bits resolution analog/digital converter, at a sampling rate of 128 Hz. The stored EEG signals were segmented into non-overlapping 2.048s epochs and were carefully inspected offline to eliminate artifacts. Absolute power was obtained for each epoch using the program POTENCOR [25]. Absolute power values were averaged over frequency bins to obtain the following broad bands: delta (1.5–3.5 Hz), theta (3.5–7.5 Hz), alpha1 (7.5–9.5 Hz), alpha2 (9.5–12.5 Hz), beta1 (12.5–17.5 Hz) and beta2 (17.5–30 Hz). The resulting values were then averaged over all epochs for each period of the cycle and for each subject. Note that this time span has a total number of 10 epochs for each subject of lengths 2, 4, 1, 5, 1, 6, 1, 3, 1, and 3 days respectively. The absolute power of the brain signals, as the described EEG recording delivered them, was obtained during the days 1, 3, 7, 8, 13, 14, 20, 21, 24 and 25, corresponding to a cycle. Table 1 shows the average of these absolute powers for each one of the brain signals. Table 2 shows the values of progesterone and estrogen levels during 28 days in an arbitrary scale convenient for the Fourier analysis.

**Table 1:** Primary absolute average power brain signals, as the EEG delivered them, from the nine young women.

Brain signals (Hz) (absolute power)										
Day	1	3	7	8	13	14	20	21	24	25

Delta	388.7	399.0	336.8	402.6	366.8	376.2	388.6	375.3	382.8	396.3
Theta	374.5	359.4	362.9	368.5	339.6	346.9	384.9	320.9	373.4	363.7
Alpha1	378.6	329.6	417.3	323.5	331.5	283.6	413.0	304.4	390.7	360.0
Alpha2	230.5	231.3	194.6	270.3	256.4	345.1	262.5	264.7	274.5	392.1
Beta1	137.0	154.8	129.4	137.5	126.8	134.2	135.6	145.7	160.4	130.5
Beta2	172.5	167.1	156.6	159.0	139.3	144.2	146.4	170.6	213.5	140.8

Menstrual cycle days are divided as follows: 1,3: Menstrual phase; 7,8: Follicular phase; 13,14: Ovulatory phase; 20,21: Early luteal phase; 24,25: Late luteal phase.

**Table 2:** Progesterone and estrogen levels rescaled in appropriate units suitable for the described Fourier analysis through 28 days.

Hormone/day	1	2	3	4	5	6	7	8	9	10
Progesterone	0.04	0.04	0.15	1.15	0.90	0.03	0.13	0.40	0.13	0.64
Estrogen	0.65	0.80	0.20	0.20	0.20	0.20	0.65	0.55	0.70	0.60

Hormone/day	11	12	13	14	15	16	17	18	19	20

Progesterone	0.35	0.15	0.50	0.15	0.05	1.35	0.85	2.15	3.00	5.00
Estrogen	0.75	1.00	2.40	3.10	2.30	1.90	1.10	0.50	0.85	0.65

Hormone/day	21	22	23	24	25	26	27	28		

Progesterone	4.40	4.65	3.18	2.00	1.05	0.65	0.35	0.15		
Estrogen	1.40	1.05	1.00	1.05	1.00	1.35	0.40	0.35		

### The discrete Fourier transform

We present here the method of analysis used to study the brain waves and sex hormone levels. Our discrete Fourier transform is presented in this section and proofs and examples are given elsewhere [20,21,26]. This nonstandard Hermite-Gaussian quadrature yields a truly discrete Fourier transform which can be used to compute the spectrum of a sampled nonperiodic (or periodic) signal and to break it down into their sinusoidal components of specific frequencies. Some of the numerical examples given in [20,26] deal with singular or oscillatory signals for which our discrete Fourier transform performs well, whereas the usual discrete Fourier transform, i.e., the so-called Fast Fourier Transform, fails to yield the correct spectrum. Our discrete Fourier transform can also be used in the frequency analysis of transient signals, like those appearing in some voice recognition problems [27].

Let us denote by $\hat{f}$(*ω*) the Fourier transform of the function *f*(*t*) defined by

(1) $$\hat{f}(\omega )=\int_{-\infty }^{\infty }e^{i\omega t}f(t)dt,$$

and let *H*_*N*_(*t*) and *t*_1_, *t*_2_,...,*t*_*N *_be the *N*^*th *^Hermite polynomial and its *N *zeros, respectively. Then, if *f*(*t*) is a function satisfying certain integrability conditions, the following quadrature formula holds:

(2) $$\begin{array}{cc} \hat{f}(\omega_{j})=\int_{-\infty }^{\infty }e^{i\omega_{j}t}f(t)dt=\sum_{k=1}^{N}F_{jk}f(t_{k})+R_{j}, & j=1,2,...,N. \end{array}$$

Here, *ω*_*j *_is again a zero of *H*_*N*_(*t*) and its numerical value corresponds to *t*_*j*_, i.e., *ω*_*j *_= *t*_*j*_, and

(3) $$F_{jk}=\frac{\sqrt{2\pi }{\left(-1\right)}^{j+k}2^{N-1}(N-1)!}{NH_{N-1}(\omega_{j})H_{N-1}(t_{k})}\sum_{l=0}^{N-1}\frac{i^{l}}{2^{l}l!}H_{l}(\omega_{j})H_{l}(t_{k}).$$

The component *R*_*j *_stands for the error obtained when the integral transform is substituted by the sum of the right-hand side. Note that (2) can be written in matrix form as

(4) $$\hat{f}=Ff+R,$$

where $\hat{f}$ and *f *are the vectors whose elements are given by the Fourier transform $\hat{f}$(*ω*) and the function *f*(*t*) evaluated at the zeros of *H*_*N*_(*t*) respectively, *F *is the matrix whose elements are given by (3) and *R *is the residual vector whose elements are of order O(1/*N*). This upper bound for the error takes into account critical cases where the signal to be transformed may have singularities or a slow decay at infinity. Thus, *F *is a matrix representing the Fourier transform in a vector space of finite dimension.

### Analysis of biological signals

Let *f*(*t*) be one of the signals studied here. Since the sex hormones levels can be represented by periodic functions of 28-day period and our aim is to find correlations between these and the brain signals, we assume that any signal *f*(*t*) is a 28-day periodic function which is sampled at *M *time values (days) 0 <*τ*_1_, *τ*_2_,...*τ*_*M *_< 28. The first part of the analysis of the signals (basically the calculation of the Fourier transform $\hat{f}$(*ω*) of *f*(*t*)) was carried out according to the following steps:

1. Select a number *N *of zeros of a Hermite polynomial. It should be chosen great enough to reduce the residual vector *R *but small enough to compute the matrix *F *with significant numerical precision.

2. Compute the Fourier matrix *F*.

3. Interpolate the *M *values *f*(*τ*_*k*_) with a trigonometric polynomial.

4. Shift and scale the interpolated function to the interval [-*π*, *π*] to yield a 2*π*-periodic signal $\tilde{f}$(*t*) (this step is not necessary, but convenient from the numerical point of view).

5. Evaluate the polynomial $\tilde{f}$(*t*) obtained in the previous step at the *N *zeros of Hermite to yield the vector *f*.

6. Compute the Fourier vector through the relation $\hat{f}$ = *Ff*.

7. Interpolate to a trigonometric polynomial $\hat{f}$(*ω*) the values $\hat{f}$_*k*_.

Basically, the sequence of analysis is: first, interpolate the samples to give a periodic function and second, break this function down into their sinusoidal components through our discrete Fourier transform.

The second part of the analysis was done according to the following. Since the Fourier transform $\hat{f}$(*ω*) of the signal is composed by a real and an imaginary part, it is necessary to determine if one of them or both are important for the frequency analysis. Thus, we first compute the Fourier transforms of each signal and take apart the real and imaginary components. Concerning the brain signals, it comes out that the values of the real part of the transformed signals at *ω *≠ 0 are all negligible compared with the great values they take at *ω *= 0. This behavior, which is typical of the Fourier Transform of a constant function, is common to all the real part of the transformed signals, whereas the imaginary part varies significantly among the different brain signals. Thus, the real part of a transformed signal is more related to the power delivered than the imaginary part, which is more related to the frequency dependence of the signal. Therefore, only the imaginary part of the transformed brain signals will be taken into account in our analysis. On the other hand, in the case of the Progesterone and Estrogen levels, the real part of the Fourier Transform comes out as important as the imaginary part, consequently both of them are taken into account.

Each signal was normalized to the height of its biggest peak. This is not necessary for Fourier analysis, but it is convenient to make uniform the data in order to compare them. Clearly, the coincident maxima define common frequencies for some signals at frequencies 1, 2, 3 and 4 units, or equivalently, at periods of 28, 14, 10 and 7 days (A frequency unit means that the signal will repeat itself each 28 days, a higher frequency means a shorter period). For coincident frequencies we will analyze only those frequencies where the value of the transformed signals are above 1/3. This percentile value gives a criterion for what "dominant" means and in this way we safely cut out all the contribution of the exposed technique to the data background, ensuring that we treat mainly the signal of interest.

There appear dominant frequencies (A word of caution is important. These frequencies correspond to the main sinusoidal components of a given signal and are related to the time needed to have that main component back again. The EEG frequency is of different nature). Some of them coincide, in particular, for a brain signal and one of the hormones. A pair of signals (or more) centered around a characteristic frequency of interest is taken into account in our study only if both of them have the value 1/3 at least, as already mentioned. This condition seems arbitrary, however the obtained results are plausible. For values smaller than 1/3, signals probably arise from noise generated during the discrete Fourier transform. The results correspond to trigonometric functions which main components present a novel and interesting relative phase. Therefore, signals with equal periods present, in general, a phase between them that can be estimated directly from a simple plot. In order to get the relative phase between two coincident periods, we consider only the values around the desired frequency. These windowed signals are then back-transformed to the time variable to get their relative phase by comparing the corresponding Fourier components. By this procedure, the EEG recording and progesterone and estrogen levels are characterized and related through the use of the Fourier transform we have selected.

## Results

**Figure 1:**
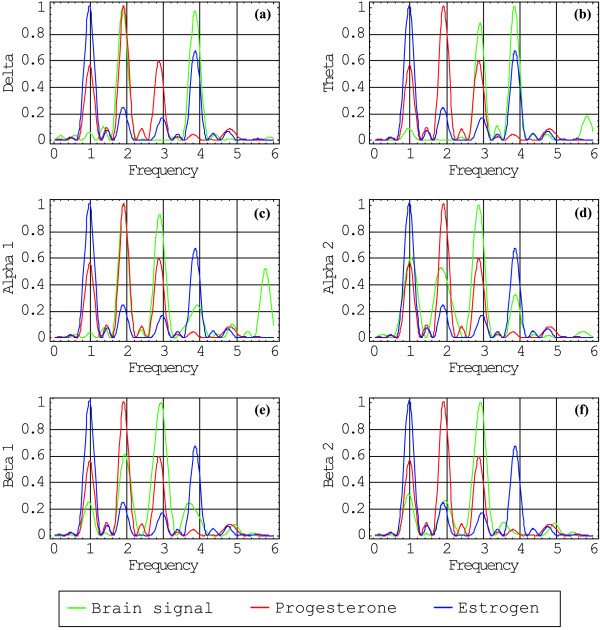
**Intensity of the different brain signals superimposed to hormone level signals**. The horizontal scale is 2*π*/28 days^-1^. The vertical scale has normalized arbitrary units. It is shown period coincidences and relative intensities in different points; when the peaks agree and both are bigger than 1/3 the coincidences were taken into account for this analysis, considering the smaller peaks as noise generated from the discrete Fourier transform.

**Figure 2:**
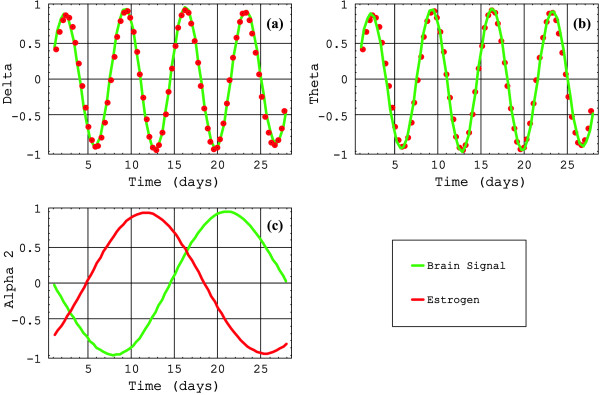
**Estrogen signals versus brain signals**. Red dots and red continuous lines are used indistinctly to clarify the figures. Delta and theta are in phase with estrogen signal; alpha2, in relative phase of 10 days.

**Figure 3:**
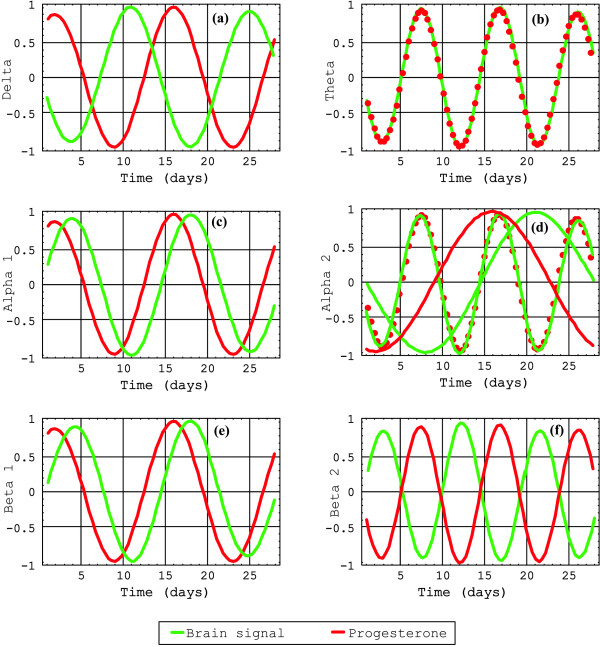
**Progesterone signals versus the six brain signals**. Different relative phases are presented between brain signals and progesterone signal.

The transformed signals are plotted in Figure 1 against frequency in units of 2*π*/28 days^-1^. The frequency patterns for each one of the EEG-signals and the two hormone levels are plotted below for positive values of the frequencies. As it can be seen, each one of these eight signals presents a peculiar pattern, a characteristic spectrum, each one in different color and normalized to 1. In blue are plotted the estrogen signals; in red, the progesterone signals; in green, the brain signals. In (a), according to the selection criterion, the spectrum of the delta brain signal versus estrogen shows a coincidence in 4; versus progesterone, a coincidence in 2. In (b), the spectrum of the theta brain signal versus estrogen shows a coincidence in 4; versus progesterone, a coincidence in 3. In (c), the spectrum of the alpha1 brain signal versus estrogen shows non coincidence; versus progesterone, a coincidence in 2 and 3. In (d), the spectrum of the alpha2 brain signal versus estrogen shows a coincidence in 1; versus progesterone, a coincidence in 1, 2, and 3. In (e), the spectrum of the beta1 brain signal versus estrogen shows non coincidence; versus progesterone, a coincidence in 2 and 3. In (f), the spectrum of the beta2 brain signal versus estrogen shows non coincidence; versus progesterone, a coincidence in 3. The Fourier transform of each brain signal and hormone levels were plotted superimposed each other, to analyze relative phases between them. When one of the relevant brain signal periods essentially coincides with one of the hormone levels, the relative phase between the two corresponding time functions is displayed in Figure 2 and Figure 3. It should be remarked that the phases presented in each one of these figures correspond to the relevant trigonometric components of the whole signal, centered around the frequency of interest, for we have related coincident relevant frequencies of the two signals considered.

In Figure 2(a), the estrogen levels and delta, with 7-day period, show a relative phase of 0 days. The estrogen levels and theta, with 7-day period, in (b), show a relative phase of 0 days. The estrogen levels and alpha2, with 28-day period, in (c), show a relative phase of 10 days. In Figure 3, the progesterone levels and delta, 14-day period, in (a), show a relative phase of 5 days. The progesterone levels and theta, 10-day period, in (b) show a relative phase of 0 days. The progesterone levels and alpha1, 14-day period, in (c), show a relative phase of 2.5 days. The progesterone levels and alpha2, 10-day period and 28-day period, in (d), show a relative phase of 0 days and 5 days respectively. The progesterone levels and beta1, 14-day period, in (e), show a relative phase of 2.5 days. The progesterone levels and beta2, 10-day period, in (f), show a relative phase of 5 days. The phases are directly taken from the figures.

## Discussion

We have presented a proposal to analyze long-period biological signals. In the cases studied here, two or more biological signals present nearly coincident frequencies and, consequently, equal periods. We also obtained the relative phases between two or more of these coincident signals. The most relevant frequency components of each signal represent an important part of the total power of this signal. Since each one of these components represent an important part of the whole signal, this behavior seems to indicate the degree in which certain brain signal couple to a specific sex hormone during the 28-day period. In the cases presented here, the brain signals delta and theta seem to accompany the estrogen, i.e., they are in phase all the time, however, alpha2 acts against to the estrogen evolution, i.e., they are out of phase all the time. Theta, alpha1, and beta1 evolve essentially as the progesterone, whereas delta and beta2 go in the contrary sense, and alpha2 follows progesterone, presenting in one case a difference of phase.

It is worth to notice that the reliability of our results was tested by adding some noise to the measured data and then carrying out the same analysis used for the unmodified signals. As expected, the analysis of the altered signals comes to different conclusions, pointing to the robustness and reliability of the method under consideration in this paper. Also notice that the method followed here is more deterministic than statistical: once the averaged signals are given, the conclusions can be drawn directly.

The reliability of repeated EEG recordings has been previously studied along 28 days [28] and considering also nine months [7]. The EEG data utilized in the present study [17] was taken and processed under similar conditions. Our analysis for long time periodic signals (28 days), as mentioned, is based on these reliable data of hormone levels and brain signals. Since brain signals have been associated with functional abilities and difficulties for certain brain processes [29-31] as well as cognitive processes, particularly, associated with women sex hormones [13,14,32-34], the method presented here could be used to give additional information on the importance of sex hormones and brain signals on several functional abilities and cognitive processes. A careful study of the meaning of our results, in connection with these aspects, is beyond the scope of our present work.

## Limitations

Before ending, we would like to emphasize some assumptions and procedures to which our analysis is circumscribed.

With respect to the Fourier analysis: 1) We have assumed that both the brain signals and sex hormones levels are described by functions that repeat themselves every 28 days. This is an important assumption but usually taken by granted, 2) the method requires a pre-analysis to determine if the real part or the imaginary part (or both) of the Fourie-transformed signal should be taken into account, 3) the method requires a criterion to decide which peak of the Fourie-transformed signal corresponds to a relevant part of the signal of interest.

Concerning the measured data: 1) The EEG data utilized in this study was obtained from subjects at rest and with open eyes. This is a particular condition to take into account, 2) finally, the EEG data correspond to subjects with ages in certain range.

## Conclusion

Our findings suggest that the proposed method achieved in the dataset is useful to find a characteristic profile of coincident frequencies and relative phases among brain activity and hormonal signals. The approach and kind of analysis we introduce here could be generalized to study other biological signals and to search for possible similar patterns and relations to those shown in this work. The knowledge of the typical periods and phases among biological signals of clinical interest seems to provide us with an auxiliary tool of analysis of possible diagnostic use.

## Competing interests

The authors declare that they have no competing interests.

## Authors' contributions

SSO provided the absolute power values of the brain signals and the hormone levels data as a function of time along the 28 days, and inquired on its relation with frequencies. RGC mainly performed the discrete Fourier analysis and found the way to define periods and relative phases. JF analyzed the consistency of the data and contributed to the Fourier analysis. OO proposed the long period analysis and drafted the manuscript. All authors contributed to the final version of the manuscript and approved it.
